# Analysis of the Ex Vivo and In Vivo Antiretroviral Activity of Gemcitabine

**DOI:** 10.1371/journal.pone.0015840

**Published:** 2011-01-14

**Authors:** Christine L. Clouser, Colleen M. Holtz, Mary Mullett, Duane L. Crankshaw, Jacquie E. Briggs, Jay Chauhan, Ilze Matise VanHoutan, Steven E. Patterson, Louis M. Mansky

**Affiliations:** 1 Institute for Molecular Virology, University of Minnesota, Minneapolis, Minnesota, United States of America; 2 Department of Diagnostic & Biological Sciences, MinnCResT Program, School of Dentistry, University of Minnesota, Minneapolis, Minnesota, United States of America; 3 Center for Drug Design, University of Minnesota, Minneapolis, Minnesota, United States of America; 4 Department of Microbiology, Medical School, University of Minnesota, Minneapolis, Minnesota, United States of America; 5 Masonic Cancer Center Comparative Pathology Shared Resource, University of Minnesota, Minneapolis, Minnesota, United States of America; 6 Department of Food Science and Nutrition, VA Medical Center, Minneapolis, Minnesota, United States of America; Massachusetts General Hospital, United States of America

## Abstract

Replication of retroviral and host genomes requires ribonucleotide reductase to convert rNTPs to dNTPs, which are then used as substrates for DNA synthesis. Inhibition of ribonucleotide reductase by hydroxyurea (HU) has been previously used to treat cancers as well as HIV. However, the use of HU as an antiretroviral is limited by its associated toxicities such as myelosuppression and hepatotoxicity. In this study, we examined the ribonucleotide reductase inhibitor, gemcitabine, both in cell culture and in C57Bl/6 mice infected with LP-BM5 murine leukemia virus (LP-BM5 MuLV, a murine AIDS model). Gemcitabine decreased infectivity of MuLV in cell culture with an EC50 in the low nanomolar range with no detectable cytotoxicity. Similarly, gemcitabine significantly decreased disease progression in mice infected with LP-BM5. Specifically, gemcitabine treatment decreased spleen size, plasma IgM, and provirus levels compared to LP-BM5 MuLV infected, untreated mice. Gemcitabine efficacy was observed at doses as low as 1 mg/kg/day in the absence of toxicity. Higher doses of gemcitabine (3 mg/kg/day and higher) were associated with toxicity as determined by a loss in body mass. In summary, our findings demonstrate that gemcitabine has antiretroviral activity ex vivo and in vivo in the LP-BM5 MuLV model. These observations together with a recent ex vivo study with HIV-1[1], suggest that gemcitabine has broad antiretroviral activity and could be particularly useful in vivo when used in combination drug therapy.

## Introduction

Retroviruses are a significant source of morbidity and mortality worldwide. For example, there are 33 million people infected with human immunodeficiency virus type 1 (HIV-1) whereas the retrovirus, xenotropic murine leukemia virus like-virus (XMRV), has recently been proposed to be linked to prostate cancer and chronic fatigue syndrome (CFS) [2], [3]. Although there are a number of drugs available for HIV-1 chemotherapy, the efficacy of these treatments is limited by the emergence of drug resistance, cost of treatment, and off-target effects. These limitations necessitate the development of new drugs and novel drug targets for HIV as well as other retroviruses. Similarly, if XMRV is shown to be the etiological agent of either prostate cancer and/or CFS, the development of new drugs could reduce morbidity and mortality.

Current anti-retroviral drugs target viral proteins that are necessary for viral replication and production. Under suboptimal therapy, the combination of both the high rates of replication and mutation leads to the emergence of drug resistance. Although drugs that target host proteins could delay or prevent the emergence of drug resistance, there are significant side effects associated with inhibiting host proteins. While cellular deoxynucleoside triphosphates (dNTPs) are necessary for host cell function, even small changes in dNTP pools appear to affect viral replication without significant cellular toxicity [4].

Previous studies have shown that hydroxyurea, which inhibits the cellular enzyme ribonucleotide reductase effectively decreases replication of HIV-1 and was recently shown to also inhibit hepatitis C virus replication as well [5], [6], [7]. Nonetheless, several factors make hydroxyurea undesirable as an antiviral including 1) pharmacokinetics of hydroxyurea vary from person to person making its plasma levels unpredictable and 2) significant toxicities (pancreatitis, hepatotoxicity) are associated with the use of hydroxyurea when used to treat HIV-1 infection. The toxicity of hydroxyurea is further emphasized in the murine AIDS (MAIDS) model in which all animals treated with hydroxyurea died from drug-related toxicities [8].

Alternatives to hydroxyurea that have a more desirable pharmacokinetic profile and lower toxicity concerns may offer a new and useful treatment for retroviral infections. In fact, other ribonucleotide reductase inhibitors have been shown to be more effective and less toxic in the MAIDS model [9]. However, no follow up studies have been published regarding their potential for clinical use.

2′,2′-Difluoro-2′-deoxycytidine, commonly referred to as gemcitabine represents a potential alternative to hydroxyurea as it has been shown to have two mechanisms of action including inhibition of ribonucleotide reductase [10], [11]. Gemcitabine is clinically-approved for cancer therapy and its anti-cancer mechanism is attributed to its ability to inhibit ribonucleotide reductase thereby limiting dNTP pools available for DNA synthesis in cancer cells. Since retroviruses may be more sensitive to dNTP pool alterations than cellular polymerases, we hypothesized that gemcitabine would be an alternative to hydroxyurea that could be translated to clinical use for the treatment of retroviral infections when used in combination with current anti-retroviral therapies.

In a recent study, we identified two clinically-approved drugs, decitabine and gemcitabine, that had potent anti-HIV activity in cell culture. The potency by which gemcitabine inhibited infectivity suggested that it may be useful for the treatment of retroviral infections when used with decitabine or in combination with current antiretroviral therapies.

In this study, we examined the ability of gemcitabine to inhibit replication of another retrovirus, murine leukemia virus in cell culture. Additionally, we examined the efficacy and toxicity of gemcitabine in vivo using LP-BM5 murine leukemia virus (LP-BM5 MuLV, a murine AIDS model). We chose this animal model as it has been used extensively to screen potential anti-HIV drugs and has been validated with a number of clinically approved anti-HIV drugs such as AZT and PMPA (tenofovir) that have relatively broad antiretroviral activity [12], [13], [14]. In this study, we show that gemcitabine decreased infectivity of MuLV in cell culture with no detectable cytotoxicity. Similarly, gemcitabine decreased disease progression in the MAIDS model at non-toxic doses although toxicities were detected at doses just three times that of the effective dose. Our findings, along with previous observations ([1]), indicate that gemcitabine has relatively broad antiretroviral activity with minimal toxicity and could be useful for in vivo antiretroviral combination therapy.

## Results

### Gemcitabine inhibits MuLV in cell culture

**Figure 1:**
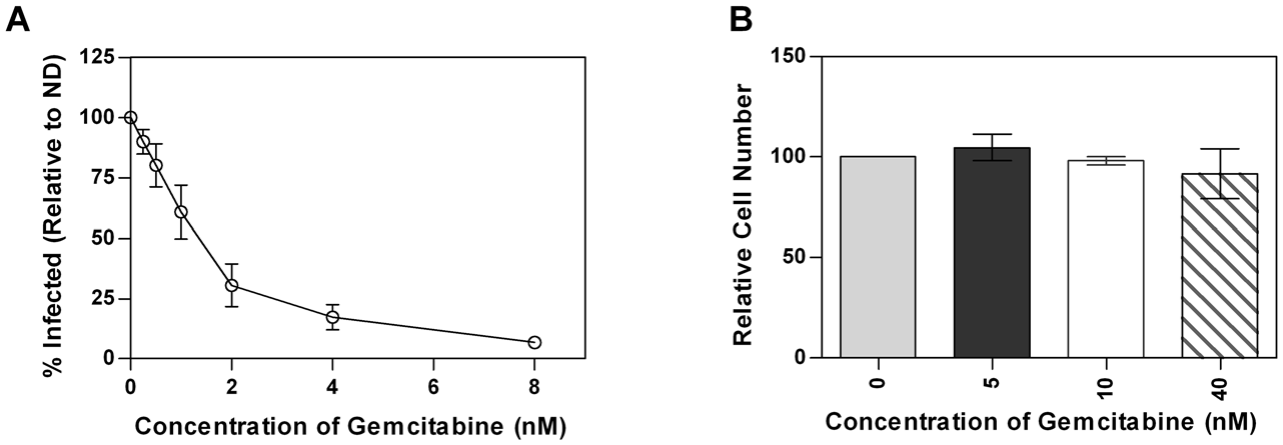
Gemcitabine inhibits MuLV replication in cell culture in the absence of toxicity. **1A**. Infectivity of MuLV. MuLV containing GFP were produced from 293T cells and used to infect U373-MAGI-CXCR4_CEM_ cells that were treated with the indicated concentrations of gemcitabine. The data represents the average ± SD of three independent experiments. **1B**. Toxicity of gemcitabine in U373-MAGI-CXCR4_CEM_ cells treated with the indicated concentrations of gemcitabine. The data represents the average ± SD of three independent experiments.

Before examining the antiviral activity of gemcitabine *in vivo*, we first examined the ability of gemcitabine to inhibit MuLV in cell culture. To do this, a GFP-tagged MuLV was pseudotyped with VSV-G and used to infect target cells that had been pretreated with increasing concentrations of gemcitabine. Flow cytometry was then used to determine the percentage of infected cells. As shown in Fig. 1A, gemcitabine potently decreased MuLV infectivity in a concentration-dependent manner with an EC50 in the low nM range. Additionally, there was no toxicity seen at the concentrations of gemcitabine needed to inhibit viral replication when the cells were exposed to gemcitabine for the same time as was used to assess gemcitabine's effect on infectivity (Fig. 1B).

### Gemcitabine inhibits progression of MAIDS as detected by spleen weight and histopathology

The data from Fig. 1 indicate that gemcitabine inhibits replication of MuLV in cell culture. To examine the ability of gemcitabine to inhibit MuLV in an in vivo system, we treated mice infected with LP-BM5 with increasing doses of gemcitabine (Table 1). Progression of MAIDS was characterized by extensive lymphoproliferation, splenomegaly, increased IgM levels, the development of lymphoma, and increased susceptibility to infection [15], [16], [17], [18].

**Table 1:** Treatment groups for ex vivo analysis of gemcitabine*.

Treatment Group	Infection Status	Treatment	Number in group at start of study	Number of surviving mice at end of study
1	Not infected	Saline	4	4
2	Infected	Saline	4	4
3	Infected	1 mg/kg/day gemcitabine	7	7
4	Infected	2 mg/kg/day gemcitabine	7	4
5	Infected	3 mg/kg/day gemcitabine	7	0
6	Infected	4 mg/kg/day gemcitabine	7	0
7	Not Infected	4 mg/kg/day gemcitabine	4	0

*Treatment Groups, number of animals at the start of the study and the number of animals surviving at the end of the study.

**Figure 2:**
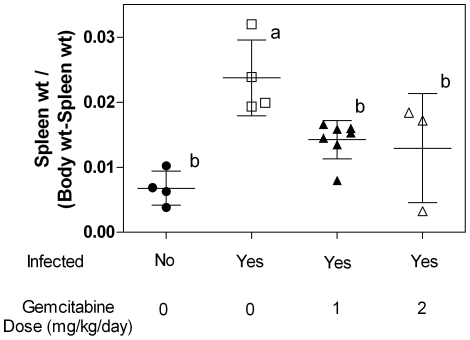
Ratio of spleen weights to body from mice infected with LP-BM5 MuLV. Each symbol (circles, squares, and triangles) represents one mouse. The average ± SD is shown. Treatment groups labeled with the same letter (eg. the 1 and 2 mg/kg/day groups are both labeled with “b”) are not statistically different from one another whereas treatment groups labeled with different letters (eg. “a” from one group and “b” for another group) are statistically different from one another as determined by One-Way ANOVA with Tukey-Kramer post-test p<0.05. n = 4 for the untreated groups, n = 7 for the mice treated with 1 mg/kg/day and n = 3 for mice treated with 2 mg/kg/day.

Proliferation of lymphoid cells in the spleen contributes to the splenomegaly observed in mice infected with LP-BM5 and is an indicator of disease progression and disease severity [18]. To determine if gemcitabine decreases splenomegaly, spleens were obtained and weighed at the time of sacrifice. As expected, the ratio of spleen to body weight was significantly increased in infected animals that did not receive treatment (Fig. 2). Mice treated with 1 or 2 mg/kg/day had an average ratio of spleen to body weight that was significantly lower than the infected, untreated mice. In fact, there was no significant difference in the spleen to body weight ratio between the treated mice and those that were not infected (Fig. 2).

Since gemcitabine significantly decreased spleen size in mice infected with LP-BM5, we next examined whether the decrease in spleen size correlated with a decrease in the histopathological changes characteristic of MAIDS. Table 2 demonstrates that there were no significant findings in spleen from two of the four uninfected mice. The spleens of the other two uninfected mice were identified as either reactive or as having a score of 1 (see Materials and Methods for scoring system). Of all other groups, the infected, untreated mice had the most severe lesions as the spleens from all four of the mice in this group received a score of 2 or higher, indicating extensive changes in splenic architecture and significant expansion of lymphoid cells. In general, there was a decrease in splenic lesion score as the dose of administered gemcitabine increased. For example, all spleens from mice receiving the highest dose of gemcitabine were normal, while 5 of the 6 mice in the 3 mg/kg/day treatment group had spleens with no significant findings and 4 of the 6 mice in the 2 mg/kg/day treatment group were within normal limits. Finally, 1 of the 7 mice from the 1 mg/kg/day treatment group had a spleen with normal histology while the other 6 spleens received a score of 1, indicating mild lesions.

**Table 2:** Histopathological analysis of spleen from gemcitabine-treated animals infected with LP-BM5.*

Not infected	Infected, untreated	1 mg/kg/day gemcitabine	2 mg/kg/day gemcitabine	3 mg/kg/day gemcitabine	4 mg/kg/day gemcitabine
NSF	2	1	NSF	NSF	NSF
NSF	2	1	NSF	NSF	NSF
R	2	1	Ab	NSF	NSF
1	3	NSF	NSF	NSF	NC
		1	NSF	NSF	NC
		1	D	Ab	NC
		1	NC	NC	NC

*Sections of spleen were analyzed as described in Materials and Methods. NSF =  no significant findings; R =  reactive, Ab = Abnormal composition; D =  depleted white pulp. NC  =  tissue was not collected. Score of 3  =  high pathology; score of 2  =  intermediate pathology; score of 1  =  low degree of pathology. Each box corresponds to a different animal.

### Treatment with gemcitabine decreases MAIDS-associated lesions in the lymph nodes

Infection with LP-BM5 destroys the architecture of lymph nodes and alters the composition [18]. When detected, the lymph nodes from infected or uninfected mice were examined by histopathology as described in the Materials and Methods section. Lymph nodes were only detectable in one of the four uninfected mice (Table 3). These lymph nodes were scored as a 1, indicating an enlargement of the lymph nodes with diffuse sheets of medium to large lymphoid cells while maintaining corticomedullary architecture. Consistent with the spleen data, the size and lesions of the lymph nodes was greatest in mice that were infected but not treated (Table 3). Additionally, lymph nodes became more difficult to detect as the dose of gemcitabine increased, indicating that gemcitabine decreased the extent of lymph node enlargement. Consistent with this, the pathology of lymph nodes from mice treated with the higher doses of gemcitabine had lower scores indicating less severe lesions. For example, 6 of the 7 mice treated with 1 mg/kg/day of gemcitabine received a score of 1, whereas all of the lymph nodes from mice treated with either 2 or 3 mg/kg/day received scores that were consistent with a decrease in disease progression when compared to the untreated but infected mice or mice treated with 1 mg/kg/day of gemcitabine.

**Table 3:** Histopathological analysis of lymph nodes from gemcitabine-treated animals infected with LP-BM5 MuLV*.

Not infected	Infected, untreated	1 mg/kg/day gemcitabine	2 mg/kg/day gemcitabine	3 mg/kg/day gemcitabine	4 mg/kg/day gemcitabine
ND	3	NSF	NSF	NSF	N/A
ND	3	NSF	NSF	NSF	N/A
ND	3	1	ND	NSF	N/A
1	2	1	ND	N/A	N/A
		1	ND	N/A	N/A
		1	N/A	ND	N/A
		2	N/A	ND	N/A

*Sections of lymph nodes were analyzed as described in Materials and Methods. NSF =  no significant findings; N/A  =  not applicable because tissues were not collected. ND  =  lymph nodes from these animals were not detected. Scores of 3  =  high degree of pathology; score of 2  =  intermediate degree of pathology; score of 1  =  low degree of pathology. Each box corresponds to a different animal.

### Effect of gemcitabine on plasma IgM levels

**Figure 3:**
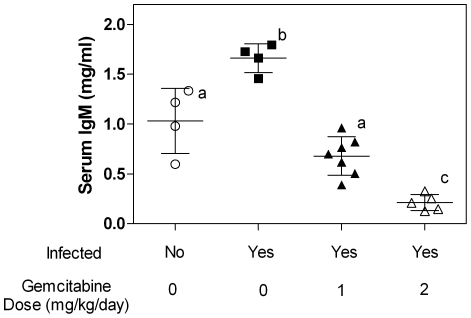
Serum IgM levels from mice infected with LP-BM5 MuLV. Each symbol (circles, squares and triangles) represent one animal. Treatment groups labeled with different letters are not statistically different from one another while treatment groups labeled with different letters are statistically different as determined by One-Way ANOVA with Tukey-Kramer post-test p<0.05. n = 4 for both the untreated groups, n = 7 for animals treated with 1 mg/kg/day, and n = 5 for animals treated with 2 mg/kg/day gemcitabine.

Soon after infection with LP-BM5, mice demonstrate a significant increase in plasma IgM levels that peak approximately 8–9 weeks post-infection [16], [18]. Subsequently, IgM levels decrease, but remain elevated compared to uninfected animals. To examine the effect of gemcitabine on IgM levels, plasma was isolated from whole blood collected from mice at the time of sacrifice. As expected, infected but untreated animals demonstrated a significant increase in IgM levels compared to the uninfected animals (Fig. 3). In contrast, treatment with 1 or 2 mg/kg/day of gemcitabine significantly decreased IgM levels compared to the untreated animals. Surprisingly, IgM levels from mice treated with 2 mg/kg/day of gemcitabine were significantly lower than that seen in the uninfected animals.

### Gemcitabine decreases provirus levels

**Figure 4:**
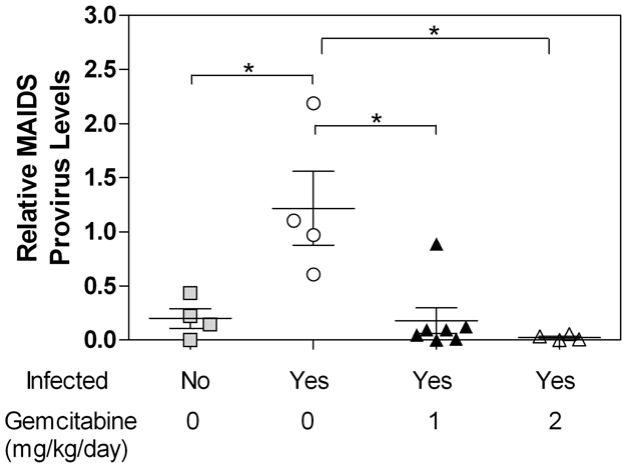
Provirus levels in mice infected with LP-BM5 MuLV. Genomic DNA was extracted from mouse spleen and quantitative real time PCR was performed to detect the defective MAIDS provirus. Provirus levels were normalized to the 18S rRNA gene. Each symbol (squares, circles, and triangles) represents one animal. The Pfaffel modification of the ΔΔCt method was used to assess gene expression. Statistical significance was assessed by One-Way ANOVA with Tukey-Kramer post-test with p<0.05 designated as significant.

The data pathology indicated that gemcitabine decreases the severity or progression of murine AIDS. However, to more directly determine if gemcitabine inhibits replication of LP-BM5, provirus levels were quantified from spleen obtained at the time of sacrifice. Levels of the defective provirus were normalized to 18S rRNA levels as previously described [19]. The data shown in Fig. 4 revealed high levels of provirus in mice that were infected, but untreated. Mice treated with either 1 or 2 mg/kg/day of gemcitabine had negligible provirus levels that were comparable to the uninfected control mice, indicating reduced viral replication.

### Toxicity of gemcitabine as determined by changes in body weight

**Figure 5:**
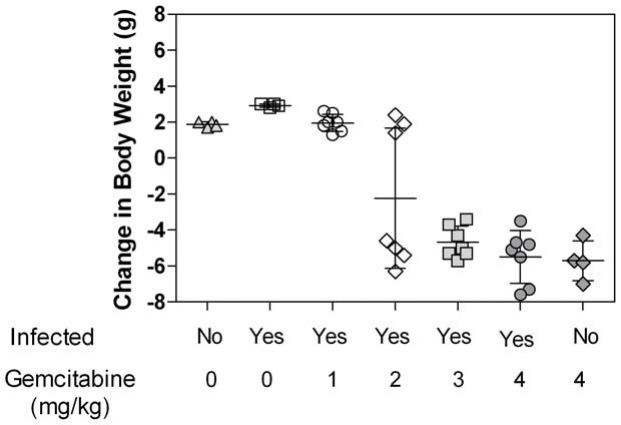
Change in body weights of mice infected with LP-BM5 MuLV and treated with the indicated doses of gemcitabine. Each symbol (triangles, squares, circles, diamonds, and hatch marks) represent one mouse. The average ± SD is shown for each treatment group.

Body weight is often used as an indicator of toxicity in laboratory animals. In this study, animals losing 15% or more of their body mass were euthanized and such loss of weight was attributed to drug-related toxicity. All mice treated with 3 and 4 mg/kg/day of gemcitabine, regardless of infection status, lost 15% or more body mass and were euthanized before the end of the study. Fig. 5 shows the change in body weight of all mice at the time of euthanasia. The data demonstrates that mice treated with 2 mg/kg/day of gemcitabine or higher lost body weight while untreated mice or mice treated with 1 mg/kg/day of gemcitabine gained body weight throughout the study.

## Discussion

In the absence of a cure or vaccine for HIV-1 infection, the identification of novel drug targets and the development of new drugs is the best approach to address the emergence of resistance as well as the complications associated with current therapies. Drugs that target cellular proteins are less likely to be susceptible to the emergence of drug resistance compared to the current anti-HIV therapies which target viral proteins. However, drugs that target cellular proteins are likely to be associated with an increase in toxicity which may limit their clinical use. For example, hydroxyurea has been used alone and in combination with nucleoside reverse transcriptase inhibitors (NRTIs) to decrease viral loads in HIV-1 infected individuals. The side effects associated with hydroxyurea has significantly curtailed its clinical use. Less toxic alternatives to hydroxyurea would offer important alternatives for the treatment of HIV-1 as well as other retroviral infections.

A recent study demonstrated that two clinically approved drugs, decitabine and gemcitabine have potent anti-HIV activity in cell culture when used alone or in combination [1]. Importantly, based on gemcitabine's potency and lack of toxicity, the data suggest that gemcitabine may be a clinically relevant alternative to hydroxyurea for the treatment of retroviral infections.

In this study, we examined the antiretroviral activity of gemcitabine in vivo and ex vivo using the LP-BM5 MuLV model (a murine AIDS model). Murine AIDS is caused by a combination of three murine leukemia viruses including BM5-eco, BM5def, and mink cell focus inducing virus (MCF) [20], [21]. BM5def is a retrovirus that is unable to replicate due to deletion of most of the pol gene. However, its intact gag gene is thought to be responsible for MAIDS pathogenesis. Although LP-BM5 is not a perfect model for AIDS pathogenesis, it is well characterized, safe, inexpensive, and has been validated with a number of clinically approved anti-HIV-1 drugs and has been useful for identifying drugs with broad antiretroviral activity (for review see [12].

Our studies found that gemcitabine decreased replication of MuLV in cell culture as well as in vivo using the MAIDS model. In fact, all measures of MAIDS-associated pathogenesis including splenomegaly, histopathology of spleen and lymph nodes and levels of IgM and provirus indicated reduced viral replication. The reduction of provirus by gemcitabine further supported the gemcitabine-mediated reduction in viral replication. There are at least two mechanisms that could account for the decrease in disease progression. First, gemcitabine could decrease dNTP levels enough to decrease proliferation of infected cells. Second, gemcitabine could alter dNTP levels such that viral replication is inhibited without inhibition of cellular proliferation. These mechanisms are likely to be dose dependent with low gemcitabine doses preferentially inhibiting viral replication and higher doses inhibiting both viral replication and cell proliferation. In support of this, 2 mg/kg/day of gemcitabine caused a significant reduction in IgM levels compared to the uninfected animals. This is likely due to gemcitabine-mediated decrease in B cell proliferation. In contrast, IgM levels from mice treated with 1 mg/kg/day had levels comparable to the uninfected animals which suggest there was not a significant reduction in B cell numbers compared to the uninfected mice. The cell culture data also supported this assertion as the decrease in replication was not associated with a significant loss of cell numbers (Fig. 1B). These data indicate that gemcitabine's antiviral activity is distinct from its activity used to treat human cancers. Furthermore, doses of gemcitabine used clinically in cancer treatment are significantly higher than those used in our study. For example, gemcitabine's anti-cancer activity is achieved with a dosing regimen that includes 1000 mg/m^2^ given once every week for seven weeks, followed by one week without drug and additional rounds of treatment as needed [22]. In our study, an antiviral effect was seen in animals at doses as low as 1 mg/kg which correlates to 3 mg/m^2^ when using the BSA method to convert the mouse dose to the human equivalent dose (mouse dose multiplied by mouse km of 3) [23]. Although the metabolism of gemcitabine in mice is likely different compared to humans, the significant difference in dosing further supports that the antiviral activity seen here is not due to inhibition of cell proliferation.

Toxicity of gemcitabine was significant at higher doses. In fact, all mice treated with 3 and 4 mg/kg/day lost 15% or greater of their body weight during the study and as a result were prematurely euthanized. However, the 1 mg/kg/day dose of gemcitabine decreased disease pathology with no detectable toxicity. Specifically, all of these animals gained body weight similar to the uninfected animals. Additionally, these animals did not show any signs of hepatotoxicity as detected by histopathological analysis (data not shown). The 2 mg/kg/day dose was also effective at decreasing disease progression. However, some of the mice in this treatment group showed signs of toxicity in the loss of body weight while others in this group appeared to tolerate gemcitabine well. Like the animals treated with 1 mg/kg/day gemcitabine, there was no hepatotoxicity observed in animals treated with 2 mg/kg/day gemcitabine. The extent to which gemcitabine effected the animals in the 2 mg/kg group was not limited to toxicity as the efficacy of gemcitabine's antiviral activity also varied (see Figure 2). Although it is not clear why the mice could have such different responses to the drug, the affect of this variation could be minimized by increasing the number of animals in each treatment group.

Although gemcitabine has been used as cancer chemotherapy for many years, it has likely been neglected as an antiviral due to its poor oral bioavailability, which would necessitate drug delivery by injection. However, a prodrug for gemcitabine has been described recently, making gemcitabine a more attractive candidate for use in treatment of retroviral infections, including highly drug-resistant HIV-1 [24]. If its bioavailability is improved, gemcitabine could serve as a novel anti-HIV drug for those resistant to the current therapies.

In summary, the findings of our study indicate that gemcitabine has potent antiretroviral activity in vivo and ex vivo using the LP-BM5 MuLV model. These findings, along with previous ex vivo HIV-1 studies with gemicitabine an decitabine, suggest that gemcitabine has broad antiretroviral activity and could be particularly useful in vivo when used in combination drug therapy.

## Materials and Methods

### Materials

SC-1/MuLV LP-BM5 cells, chronically infected with LP-BM5, were obtained through the NIH AIDS Research and Reference Reagent Program, Division of AIDS, NIAID, NIH from Dr. Herbert Morse and Dr. Janet Hartley [25], [26], [27]. 293T cells were obtained from American Tissue Type Culture (ATCC). C57Bl/6 mice were purchased from Jackson Laboratories. DMEM was purchased from MediaGrow. Gemcitabine was obtained from Carbosynth (Berkshire, U.K.). The IgM Elisa kit was from Assay Designs (Ann Arbor, MI). The plasmids, pCR-DEF and pCR-18S were a kind gift from Dr. Mauro Magnani (University of Urbino, Urbino, Italy) and have previously been described [28]. U373-MAGI-CXCR4_CEM_ cells were obtained from Dr. Michael Emerman through the AIDS Research and Reference Reagent Program, Division of AIDS, NIAID, NIH [29], [30]. The plasmid, pIRES2-EGFP was obtained from Clontech (Mountain View, CA). The plasmids pMIGR1, pJK3, pL-VSV-G, and CMV-Tat were kind gifts from Vineet Kewal Ramani (NCI-Fredrick). The plasmid pMIGR1 is an MLV vector containing an IRES-GFP element [31]; pJK3 contains GagPol driven off HIV-1 LTR [32]. pL-VSV-G is also driven off the HIV-1 LTR [32]; and CMV-Tat allows transcription off the HIV-1 LTR [32].

### Cell culture

SC-1/MuLV LP-BM5 cells and 293T cells were maintained in DMEM containing 10% fetal clone 3 (FC3) serum (Hyclone, Logan, VT) and pen/strep at 37°C in 5% CO_2_. U373-MAGI-CXCR4_CEM_ cells expressing the CD4 receptor and the CXCR4 co-receptor were maintained at 37°C in 5% CO_2_ in selection media composed of DMEM with 10% FC3, 1 µg/mL puromycin, 0.1 mg/mL hygromycin, and 0.2 mg/mL neomycin.

#### Transfection of 293 T cells

pMIGR1 (13.9 µg), pJK3 (6.9 µg), pL-VSV-G (5.54 µg) and CMV-Tat (1 µg) were transfected into 293T cells using the calcium phosphate method. Forty-eight hours after transfection, cell culture supernatant was collected and frozen at −80°C.

#### Drug Treatment/Flow Cytometry

U373-MAGI-CXCR4_CEM_ cells (62,000) were plated in 12-well dishes 24 h prior to drug treatment. Twenty-four hours later, gemcitabine was added to the cultures at the concentrations indicated in the legend to Fig. 1. Two hours post-drug treatment, 500 µl of virus was added to each culture. Twenty-four hours later, the medium containing gemcitabine (or DMSO) was removed replaced with new media. Twenty-four hours after the media change, the U373-MAGI-CXCR4_CEM_ cells were collected and analyzed by flow cytometry to determine the percentage of cells expressing GFP. Infectivity was normalized for each individual experiment by setting the infectivity of the untreated cells to 100 for each experiment and then multiplying the data from the other individual treatments by the number used to convert the no drug treated cells to 100.

#### Cellular Proliferation

Cellular proliferation was examined using the CellTiter-Glo kit from Promega according to the manufacturer's instructions. U373-MAGI-CXCR4 cells (4,500/well) were plated in a 96-well dish 24 h prior to drug treatment. Cells were treated (or not) with gemcitabine for 24 h before proliferation was assessed by luciferase activity. DMSO was used as a control for the untreated cells. Ethanol (20%) was used as a positive control for cellular toxicity. The data were converted to relative cell number by setting the no drug treated cells at 100 for each experiment and then multiplying each other sample data by the number used to convert the no drug treated cells to 100. This conversion was normalized for differences in luciferase activity among different experiments.

#### Mice

Female C57BL/6 mice aged 8–10 wk old were purchased from Jackson Laboratories (Sacramento, California) and were housed in standard rodent shoebox caging without a filter top at 22±1°C with a 12 h light/dark cycle, 60±5% humidity, and 12 air changes/h. Mice were fed lab chow and water *ad libitum*. The experimental protocol was approved by the Department of Veterans Affairs Medical Center (Minneapolis, MN) IACUC committee (IACUC protocol number 0803A28341).

### Infection of mice with LP-BM5 MuLV

LP-BM5 was produced from confluent SC-1 cells by filtering the cell supernatant through a 0.25 µm syringe. The filtered viral supernatant was maintained at −80°C until inoculation was performed. C57BL/6 mice were inoculated with two intraperitoneal injections of 0.25 mL of viral supernatant or DMEM spaced three days apart.

### Treatment of LP-BM5 MuLV infected mice with gemcitabine

C57BL/6 mice were randomly divided into 7 groups including: 1) uninfected untreated; 2) uninfected treated with 4 mg/kg/day gemcitabine; 3) infected, untreated; 4) infected treated with 1 mg/kg/day; 5) infected with 2 mg/kg/day; 6) infected, treated with 3 mg/kg/day; and 7) infected, treated with 4 mg/kg/day. All mice were treated daily with either gemcitabine or phosphate buffered saline (PBS) for 8 wk beginning 1 wk post-infection. Animals were weighed daily to achieve proper dosing and to detect changes in body mass due to toxicity or infection. Animals did not receive drug treatment 24 h prior to sacrifice.

### Sacrifice of animals

Animals were euthanized either 8 wk post-drug treatment or when animals lost 15% or greater body mass due to toxicity associated with treatment. Animals were weighed and anesthetized with 100 mg/kg ketamine plus 10 mg/kg xylazine prior to blood collection from the submandibular vein. After euthanasia, spleens were removed, weighed, and sectioned for histopathological analysis as well as for the quantification of proviral levels. Lymph nodes, when detected, were obtained and sectioned for histopathological analysis as was liver. Necropsy was performed to assess any gross abnormalities in any organs.

### Splenic histopathology

Histological examination and scoring of spleen was performed by A.C.V.P. board certified veterinary pathologists from the University of Minnesota Masonic Cancer Center Comparative Pathology Shared Resource. The severity of lymphoproliferation within the spleen was scored based on a previously published scoring system [33], [34] with slight modifications as follows:

1) Normal spleen (N) demonstrated normal architecture and a ratio of while pulp to red pulp (W∶R) of ≤ ∼1∶1. 2) Spleens designated as reactive (R) were defined as having reactive germinal center expansion with a W∶R ratio of ∼2∶1. Germinal centers in these spleens were prominent and expanded. 3) Spleens designated as a score of 1 were characterized by more extensive germinal center expansion that those in the reactive group, with a W∶R ratio of ∼3∶1. Lymphoid nodules in these spleens were not coalescent and had prominent mantle zones. 4) Spleens designated with a score of 2 contained multifocal large lymphoid nodules that coalesced and resulted in effacement of red pulp and mantle zones. The W∶R ratio in these spleens was ∼4∶1. 5) Spleens designated as a score of 3 were characterized by extensive proliferation of medium to large lymphoid cells that nearly completely effaced the red pulp and mantle zones of the lymphoid nodules. The W∶R ratio in these spleens was >4∶1. 6) Finally, spleens defined as D were characterized by lymphoid depletion within white pulp.

### Lymph node histopathology

A scoring system for lymph nodes in MAIDS mice has not been previously reported. Scoring of lymph node lesions was similar to that described for the spleen and was done using following grading scheme: 1) Normal (N) was defined as lymph nodes demonstrating normal architecture. 2) Reactive (R) was defined as lymph nodes in which the corticomedullary architecture was maintained, but the lymphoid follicles had enlarged germinal centers 3) A score of 1 was defined by moderately enlarged lymph nodes with diffuse sheets of medium to large lymphoid cells while still maintaining some follicular structures and corticomedullary architecture. 4) A score of 2 was defined as diffusely enlarged lymph node where the corticomedullary architecture was absent, but a rim of small lymphocytes was present beneath the capsule. Finally, 5) lymph nodes designated with a score of 3 were diffusely enlarged, and contained medium to large lymphocytes that infiltrated the lymph node capsule and extended into adjacent tissues.

### Serum immunoglobulin M determination

After 8 wk of treatment, IgM levels were determined by ELISA as per the manufacturers' instructions (Assay Designs, Ann Arbor, MI). Plasma was isolated from whole blood by centrifugation at 1,000×g for 10 minutes at room temperature and stored at −80°C until the assay was performed. A standard curve was generated using a standard IgM of known concentration and a correlation coefficient (R^2^) of >0.98 was considered acceptable data for analysis. All samples were run in triplicate and to confirm reproducibility between assays, a subset of samples was run in three independent experiments.

### Determination of provirus levels from spleen

At 8 wk post-treatment, BM5d DNA content was assayed from genomic DNA isolated from spleen. Total cellular DNA was isolated using the Roche kit as per the manufacturers' instructions. BM5d DNA was quantified by real-time PCR assay as previously described [19]. The amount of BM5d DNA in spleen was calculated by interpolation of the experimentally determined plasmid standard curve and was normalized to 18S rRNA. SYBR green mastermix (Applied Biosystems) containing genomic DNA (1 µl) and 25 pM of primers. The primers used to detect BM5def were 5′ CCTTTATCGACACTTCCCTTTT 3′ and 5′ TGGCAGAGGAGGAAGGTT3′. The primers used to detects 18S rRNA were: 5′ GTAACCCGTTGAACCCCATT (forward) and 5′CCATCCAATCGGTAGTAGGG (reverse). Conditions for amplification of BM5Def and 18S rRNA included an initial heat activation of the polymerase at 95°C for 13 min followed by 40 cycles of 95°C for 15 sec and 62°C for one min. Samples were then heated to 72°C for 5 min, 95°C for 1 min prior to performing a melt curve analysis from 55°C to 95°C. Data was used for analysis only when the standard curve for each primer set yielded an R^2^ of >0.99 with an efficiency of 90–110%, single peaks were observed in the melt curves and template controls gave no detectable amplification. Proviral levels normalized to 18S rRNA levels by the Pfaffel method of quantification and were determined in triplicate for each set of reactions and three independent assays were performed with each sample.
